# Surviving Mousepox Infection Requires the Complement System

**DOI:** 10.1371/journal.ppat.1000249

**Published:** 2008-12-26

**Authors:** Elizabeth A. Moulton, John P. Atkinson, R. Mark L Buller

**Affiliations:** 1 Rheumatology Division, Department of Medicine, Washington University School of Medicine, Saint Louis, Missouri, United States of America; 2 Department of Molecular Microbiology and Immunology, Saint Louis University Health Sciences Center, Saint Louis, Missouri, United States of America; Oregon Health & Science University, United States of America

## Abstract

Poxviruses subvert the host immune response by producing immunomodulatory proteins, including a complement regulatory protein. Ectromelia virus provides a mouse model for smallpox where the virus and the host's immune response have co-evolved. Using this model, our study investigated the role of the complement system during a poxvirus infection. By multiple inoculation routes, ectromelia virus caused increased mortality by 7 to 10 days post-infection in C57BL/6 mice that lack C3, the central component of the complement cascade. In C3^−/−^ mice, ectromelia virus disseminated earlier to target organs and generated higher peak titers compared to the congenic controls. Also, increased hepatic inflammation and necrosis correlated with these higher tissue titers and likely contributed to the morbidity in the C3^−/−^ mice. In vitro, the complement system in naïve C57BL/6 mouse sera neutralized ectromelia virus, primarily through the recognition of the virion by natural antibody and activation of the classical and alternative pathways. Sera deficient in classical or alternative pathway components or antibody had reduced ability to neutralize viral particles, which likely contributed to increased viral dissemination and disease severity in vivo. The increased mortality of C4^−/−^ or Factor B^−/−^ mice also indicates that these two pathways of complement activation are required for survival. In summary, the complement system acts in the first few minutes, hours, and days to control this poxviral infection until the adaptive immune response can react, and loss of this system results in lethal infection.

## Author Summary

As one of the most successful pathogens ever, smallpox caused death and disfigurement worldwide until its eradication in the 1970s. The complement system, an essential part of the innate immune response, protects against many pathogens; however, its role during smallpox infection is unclear. In this study, we investigated the importance of the complement system in mousepox infection as a model for human smallpox disease. We compared mice with and without genetic deficiencies in complement following infection by multiple routes with ectromelia virus, the causative agent of mousepox. Deficiencies in several complement proteins reduced survival of ectromelia infection. Sera from these same complement-deficient mice also have reduced ability to neutralize ectromelia virus in vitro. In complement-deficient mice, ectromelia virus disseminated from the inoculation site earlier and produced higher levels of virus in the bloodstream, spleen, and liver. The increased infection in the liver resulted in greater tissue damage. We hypothesize that the complement-deficient mice's reduced ability to neutralize ectromelia virus at the inoculation site resulted in earlier dissemination and more severe disease. We have demonstrated that surviving ectromelia virus infection requires the complement system, which suggests that this system may also protect against smallpox infection.

## Introduction

Poxviruses remain a threat to the human population despite the eradication decades ago of naturally circulating variola virus, the causative agent of smallpox. Smallpox, with its up to 30% mortality rate, could devastate the large unvaccinated population if released accidentally or by bioterrorists [1]. Closely related monkeypox virus has also emerged as a human pathogen [2]. To understand the virulence of smallpox, investigators have turned to related poxviruses like ectromelia virus (ECTV), the causative agent of mousepox. Variola virus and ECTV have a narrow host-range and cause significant morbidity and mortality [3],[4]. The numerous available mousepox-susceptible and -resistant mouse strains allow the components of the protective immune response to poxviruses to be dissected in the natural host.

Disease severity varies among inbred mouse strains, and comparisons of these strains have elucidated factors essential for survival. Mice naturally acquire ECTV via cutaneous abrasions, which is mimicked experimentally with footpad inoculation [4]. Through this route, ECTV infection is 100% lethal in susceptible strains (BALB/c, DBA/2, and A/J) but asymptomatic in the resistant C57BL/6 strain. The C57BL/6 strain has a stronger T_H_1 type cytokine response and a more robust cytotoxic lymphocyte response than susceptible strains [5]. Lethal infection occurs in C57BL/6 mice that lack CD8^+^ T cells [6],[7], B cells [7],[8], macrophages [6], natural killer (NK) cells [9],[10], interferon (IFN)-γ [11]–[13], IFN α/β receptor [13], perforin [14],[15], and granzyme A or B [16]. Survival, therefore, requires both the adaptive and innate immune response.

The innate immune system defends the host during the early phase of an infection and shapes the adaptive response [17]–[19]. The complement system is an essential component of the innate immune system, and evidence from human disease and animal models implicates complement as a critical part of host defense against several virus families [20]–[24].

The complement system consists of cell-surface and serum proteins that interact to destroy invading microorganisms and infected host cells [19], [25]–[27]. Three distinct pathways activate this cascade: classical, lectin, and alternative (pathway diagram in the results section). Antibody binding to antigen triggers the classical pathway. Mannan-binding lectin (MBL) and related proteins recognize repetitive carbohydrate motifs on pathogens and infected cells to initiate the lectin pathway [28]. Spontaneously activated C3 initiates the alternative pathway, especially if deposited on surfaces deficient in regulatory proteins [29]. The alternative pathway also serves as a positive feedback loop by forming additional C3 convertases from the C3b produced by any pathway. All three pathways converge at the step of C3 cleavage to C3a and C3b, and they share a common terminal pathway that generates the C5a anaphylatoxin and the membrane attack complex (MAC).

Complement system activation can exert multiple antiviral effects [25],[27]. Opsonization of the virion may block attachment or promote destruction by phagocytosis. The MAC disrupts the membrane integrity of the virion or infected cells. The anaphylatoxin cleavage products, C3a and C5a, attract and activate proinflammatory and immune effector cells [30]. Finally, complement activation induces and instructs the adaptive response and augments the neutralizing activity of antibody [18], [31]–[33]. To evade these antiviral activities, viruses use multiple strategies to hinder complement activation [26],[27],[34].

In their large double-stranded DNA genomes, poxviruses encode factors that modify the immune response [35]. Study of immunomodulatory molecules has provided insights into viral pathogenesis and revealed novel facets of the host's immune response [36]–[39]. Variola virus, monkeypox virus, and ECTV each produce an orthologous complement regulatory protein that has structural and functional homology to host proteins [33], [40]–[45]. Loss of this complement regulatory protein may account for the reduced virulence seen in the West African vs. Congo basin strains of monkeypox virus [45],[46]. The limits of the monkeypox animal models, however, have made this a difficult hypothesis to test. Loss of the complement regulatory protein affects local lesion size of cowpox and vaccinia virus, but these are non-lethal infection models [33],[47]. Additionally, an incomplete understanding of the role of complement during poxviral infections has complicated the investigation into how these proteins enhance virulence.

Complement influences poxviral infections, but an essential role for survival has not been demonstrated. One study described increased inflammation at the inoculation site of cowpox virus in C5^−/−^ mice; however, no mortality occurred in these mice [48]. Additionally, an allele for genetic resistance to ECTV mapped to the chromosomal region containing C5 [49].

Using complement-deficient mice, the mousepox model offers an opportunity to characterize the role of this system during infection in the natural host. Use of a model where the host and pathogen have co-evolved is particularly important given the species specificity of many poxviruses and of complement proteins, regulators, and receptors [3],[50]. In this study, we focused on the role of C3, the complement cascade's central component. Resistant C57BL/6 mice that genetically lack C3 inadequately control ECTV infection and have increased morbidity, viral burdens, and mortality. Our in vitro and in vivo evidence suggests that the complement system neutralizes ECTV early in infection and contributes to survival.

## Results

### C3 Deficiency Increased Mortality from ECTV Inoculated by Multiple Routes

The route of infection influences the interaction between poxviruses and the host [51]. Half of the 16 mutant vaccinia viruses assessed using two routes of inoculation, ear pinna or intranasal, had a detectible phenotype by only one route. ECTV infections of C57Bl mice by the intranasal, intraperitoneal, or intravenous routes result in severe disease and mortality, while the footpad and intradermal routes cause minimal disease [52]. To examine the role of complement in vivo, wild-type and C3^−/−^ mice were infected by three routes: footpad, ear pinna, and intranasal.

**Figure 1:**
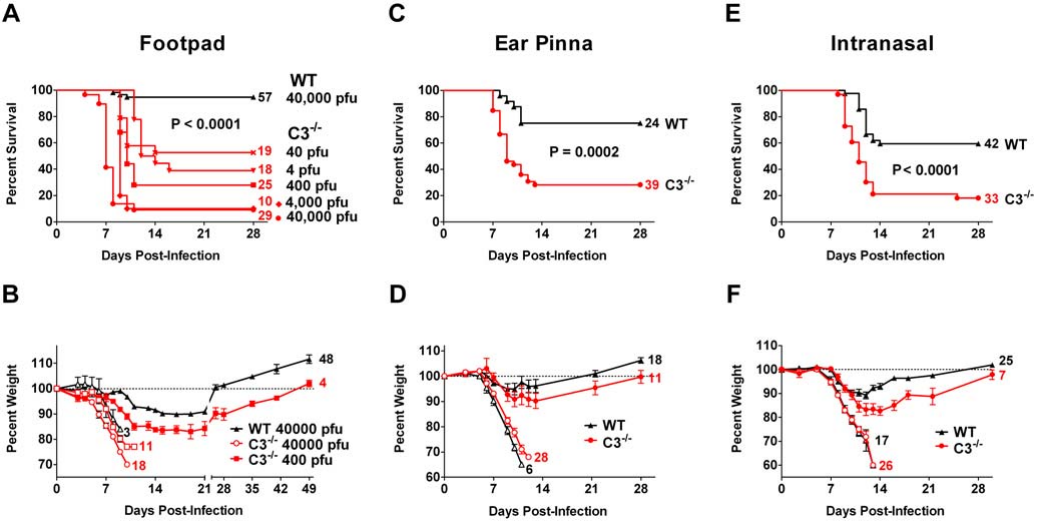
C3 deficiency increased mortality and morbidity in C57BL/6 mice. (A,C,E) Greater mortality was observed in C3^−/−^ compared to wild-type (WT) C57BL/6 mice by multiple routes of inoculation. The number of animals in each group is right of the line, and the statistics compare the survival of C3^−/−^ and wild-type mice. (B,D,F) C3^−/−^ mice have increased morbidity following infection by the indicated route as monitored by weight loss. Weight loss for individual animals was normalized to their starting weight, and mean±SEM is shown. The graph segregates the mice that survived the infection (filled symbols) from those that succumbed (open symbols). The number of animals in each group is presented at the end of the line. (A,B) Mice were inoculated in the footpad with the dose listed to the right of the survival curve or in the legend. The survival curves were generated from two to three separate experiments (except for a single experiment at 4,000 pfu). The survival of the C3^−/−^ mice significantly differed from the wild-type mice at all doses (*P*<0.0001). (B) The one C3^−/−^ mouse that survived at 40,000 pfu is not shown for clarity. C3^−/−^ mice alive at day 49 continued to gain weight until day 119 post-infection. (C,D) Mice were inoculated with ∼700 pfu ECTV via the ear pinna. The data are combined from four separate experiments (*P* = 0.0002). (E,F) Mice received 100 pfu of ECTV intranasally. The curves are generated from at least seven separate experiments (*P*<0.0001).

Approximately 95% (54 of 57) of the wild-type mice survived when inoculated with 40,000 pfu of ECTV, the highest dose employed in the footpad infections (Figure 1A). In contrast, C3^−/−^ mice had about 90% mortality at that dose. They also had significantly increased mortality (*P*<0.0001) at lower doses, even when inoculated with only 4 pfu. The median time to death increased as the dose decreased from 7 days at 40,000 pfu to 9, 10, or 13 days at the lower doses of 4,000, 400, or 4 pfu, respectively.

The C3^−/−^ mice also showed increased morbidity over the course of the infection. Unlike the wild-type mice on day 7 post-infection with 40,000 pfu, the C3^−/−^ mice displayed clinical signs of infection, including fur ruffling and hunchbacked posture. Consistent with these observations, C3^−/−^ mice lost more weight at 400 and 40,000 pfu than wild-type (Figure 1B). The few surviving C3^−/−^ mice at the 400 pfu dose required ∼3 additional weeks compared to the wild-type mice to return to their initial weight. All surviving mice in Figure 1A were held for at least 40 days to monitor recovery, and a subset of C3^−/−^ mice (*n* = 4 at 400 pfu) were held to day 119 post-infection. The mice that survived the acute illness recovered weight steadily and showed no signs of relapse.

The ear pinna studies used a dose of 700 pfu to mimic the low inoculum thought to transmit the natural poxvirus infection [2]. The infection caused 72% mortality in the C3^−/−^ mice (28 of 39) (Figure 1C), compared to 25% in the wild-type mice (6 of 24, *P* = 0.0002). The surviving C3^−/−^ mice lost less weight and recovered to the initial weight earlier if inoculated by the ear pinna compared to the footpad route (Figure 1B and 1D). In contrast to the increased morbidity and mortality observed, C3 deficiency caused no gross differences in the primary lesion; C3^−/−^ and wild-type mice had similar levels of footpad swelling or necrosis at the ear pinna inoculation site (data not shown).

To examine the role of C3 in intranasal infection, the dose was lowered to 100 pfu due to the increased susceptibility of the wild-type mice with this route. C3 deficiency increased the mortality rate from 40% to 80% (*P*<0.0001, Figure 1E). Similar to the other routes, the surviving C3^−/−^ mice had more severe disease than wild-type, as they lost more weight and took longer to recover (Figure 1F).

### C3 Deficiency Resulted in Earlier Dissemination of ECTV to the Target Organs, Higher Peak Viral Titers, and Delayed Viral Clearance

ECTV replicates at the inoculation site and in the draining lymph node to generate the primary viremia that infects the spleen and liver [4]. Virus released from these target organs causes a secondary viremia, which seeds distal sites like the skin, generating the characteristic pox lesions.

**Figure 2:**
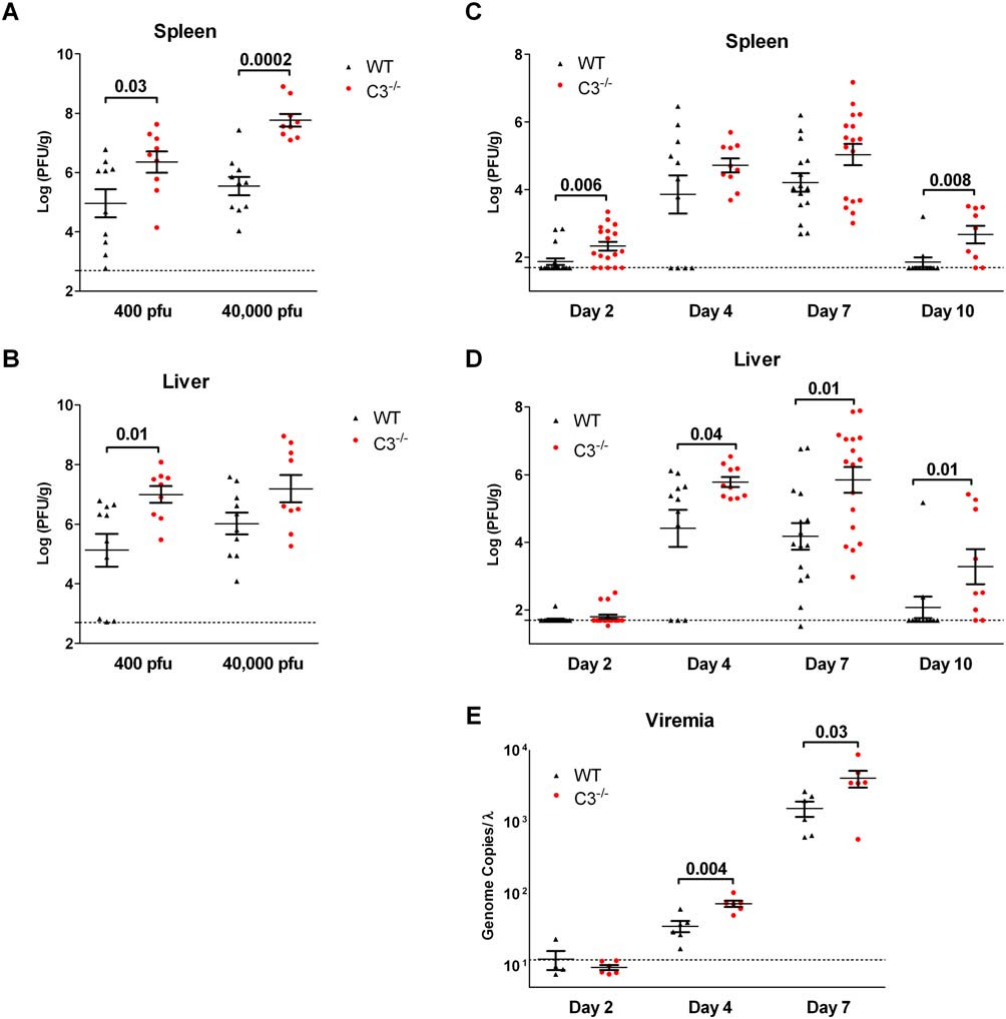
C3 deficiency promoted earlier dissemination to and increased viral titers in the target organs. C3^−/−^ and wild-type mice were infected by the footpad or ear pinna route and sacrificed on day 2, 4, 7, or 10 post-infection. The viral titer of the spleen (A,C) or liver (B,D) was determined by direct plaque assay and is expressed as the log_10_ plaque forming units (PFU)/g tissue. The level of viral DNA in whole blood was measured using quantitative PCR (E). The scatter plots show the viral burden of each wild-type (WT, black, ▴) and C3^−/−^ (red, •) animal. The error bars indicate SEM. The wild-type and C3^−/−^ mice were compared, and *P* values <0.05 are reported above the bracket. The dotted line represents the limit of detection for the assay. (A,B) Mice were infected with 400 or 40,000 pfu of ECTV in the footpad and sacrificed on day 7 post-infection. At 400 pfu, all animals had virus detected in either the spleen or liver. (C–E) Animals were infected with ∼700 pfu of ECTV via the ear pinna. Mean viral titers±SEM or genome copies±SEM from the scatter plots are also plotted against time in a line graph (Figure S1).

To begin to dissect how C3 contributed to protection against ECTV, we examined viral burden in two key tissues, the spleen and liver. Wild-type and C3^−/−^ mice were inoculated in the footpad with either 400 or 40,000 pfu, and then spleen and liver tissue were collected on day 7 post-infection. All animals had detectible virus in either the spleen or liver. At the two doses, the C3^−/−^ mice had a 1–2 log higher mean titer than wild-type mice in both tissues (Figure 2A and 2B).

In wild-type mice, both doses produced similar maximal tissue titer; however, the higher dose increased the uniformity of the group and, thereby, increased the mean titer. At the 40,000 pfu dose, the splenic viral burden in the C3^−/−^ mice was ∼150-fold higher (*P = *0.0002, Figure 2A). Reducing the dose to 400 pfu resulted in ∼25-fold lower viral titer in the C3^−/−^ mice, yet it was still ∼25-fold higher than the wild-type controls (*P = *0.03). In contrast, both doses produced similar liver titers in the C3^−/−^ mice. The lower dose revealed an 80-fold increase in the liver titer of the C3^−/−^ mice compared to the wild-type mice (*P = *0.01, Figure 2B), while the higher dose showed less of a difference between the strains (15-fold) due to the increased titer in the wild-type mice. Illustrative of the impact of C3, the C3^−/−^ mice at 400 pfu had higher titers than the wild-type mice given 40,000 pfu, a 100-fold more virus.

These increases in viral titer prompted further exploration of how C3 deficiency impacts viral spread. C3 could control viral replication early at the inoculation site by directly inactivating free virus or by recruiting inflammatory cells through release of anaphylatoxins. The lack of C3 in the blood to neutralize or opsonize the virus could also result in greater viremia, thereby producing the higher titers observed in the target organs on day 7. Alternatively, C3's well-established ability to facilitate induction of antibody and T cell responses could explain the observed difference [21], [22], [53]–[57]. To elucidate when the infections in the C3^−/−^ and wild-type mice diverge, we inoculated via the ear pinna route and examined the viral burden in the blood, spleen, and liver on days 2, 4, 7, and 10 post-infection. The ear pinna route was selected for further analysis because it is a cutaneous route of inoculation that mimics a natural infection of the epithelium where complement may promote containment.

Using whole blood enables an unbiased detection of all virus, whether free in the plasma or in infected cells. Quantitative PCR was employed to detect viral DNA in blood on days 2, 4, and 7 (Figure 2E). A few day 2 samples contained viral DNA, but most were below the detection limit. The C3^−/−^ mice had 2.0- and 2.5-fold higher levels of viral DNA than wild-type mice had on days 4 and 7 (*P = *0.004 and 0.03, respectively).

Despite the low levels of viremia on day 2, infectious virus was present in the spleen of over 70% of the C3^−/−^ mice (13 of 18) compared to 28% of the wild-type mice (5 of 18, *P* = 0.006, Figure 2C). By day 7 post-infection, the C3^−/−^ mice had 45-fold higher viral titers in the liver (*P* = 0.01, Figure 2D), and there was also a similar trend in the spleen (6-fold, *P* = 0.09). The wild-type mice regained weight starting on day 10 (Figure 1D), and by then over 80% had cleared the virus from the spleen or liver (9 of 11, Figure 2C and 2D). In contrast, less than half of the C3^−/−^ mice survived to day 10 (Figure 1C), and of these, over 75% had ongoing infection of the spleen and liver (7 of 9, *P* = 0.008 and 0.01, respectively).

In summary, C3 deficiency resulted in earlier dissemination to spleen and in higher peak titers in the liver. The viral infection also continued to day 10 in the C3^−/−^ when it had been cleared by most wild-type mice.

### C3 Deficiency Increased Hepatic Inflammation and Necrosis

In susceptible mouse strains, ECTV causes extensive hepatic and splenic necrosis [58],[59]. We compared C3^−/−^ and wild-type mice for histopathological changes in the liver on days 4, 7, and 10 post-infection.

**Figure 3:**
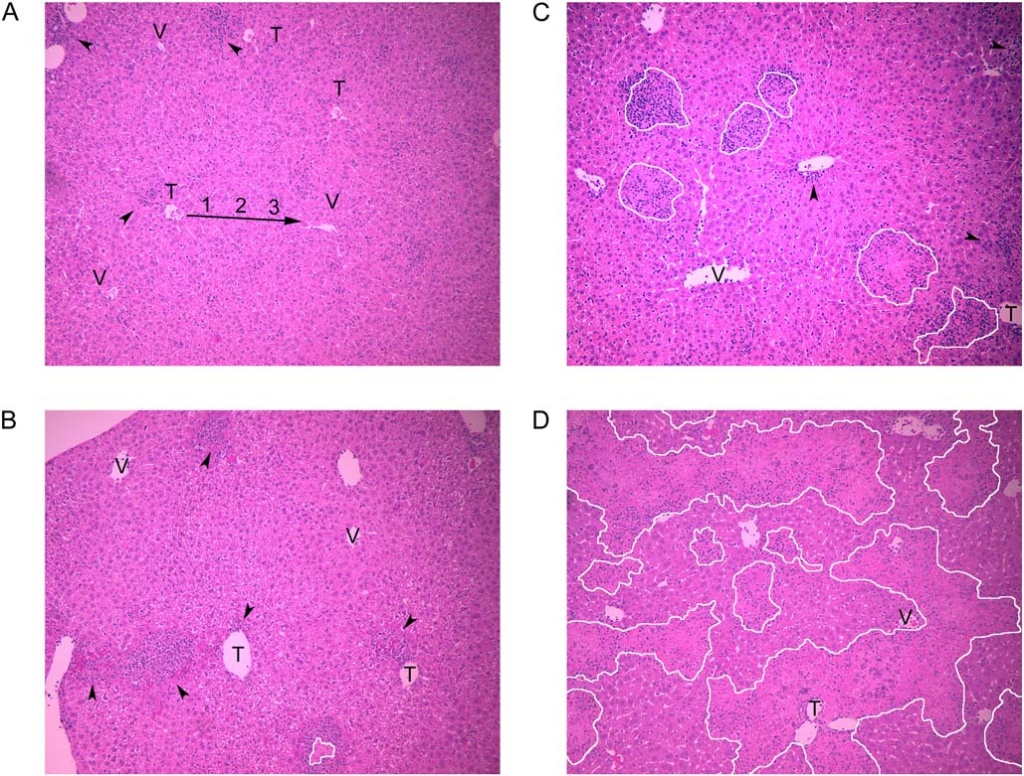
Extensive liver necrosis occurred in C3-deficient mice. Liver samples were taken from mice 7 days after infection with ∼700 pfu via ear pinna, fixed, sectioned, and stained with hematoxylin and eosin. Representative images show the range of differences between the strains. White lines border coalesced areas of necrosis. Arrowheads point to non-necrotic inflammatory foci. T, portal triad; V, central vein. (A) Wild-type—blood flows through the liver from zone 1 to 3, as indicated by the arrow. Zone 1 encircles the portal triad, zone 3 encircles the central vein, and zone 2 occurs between zones 1 and 3. There are small inflammatory foci adjacent to two portal triads. (B) Wild-type—there are inflammatory foci adjacent to portal triads, and one focus has a small area of confluent necrosis. (C) C3^−/−^—larger inflammatory foci with areas of confluent necrosis. (D) C3^−/−^—an inflammatory infiltrate borders the extensive necrosis that bridges across all three zones (T→V).

**Figure 4:**
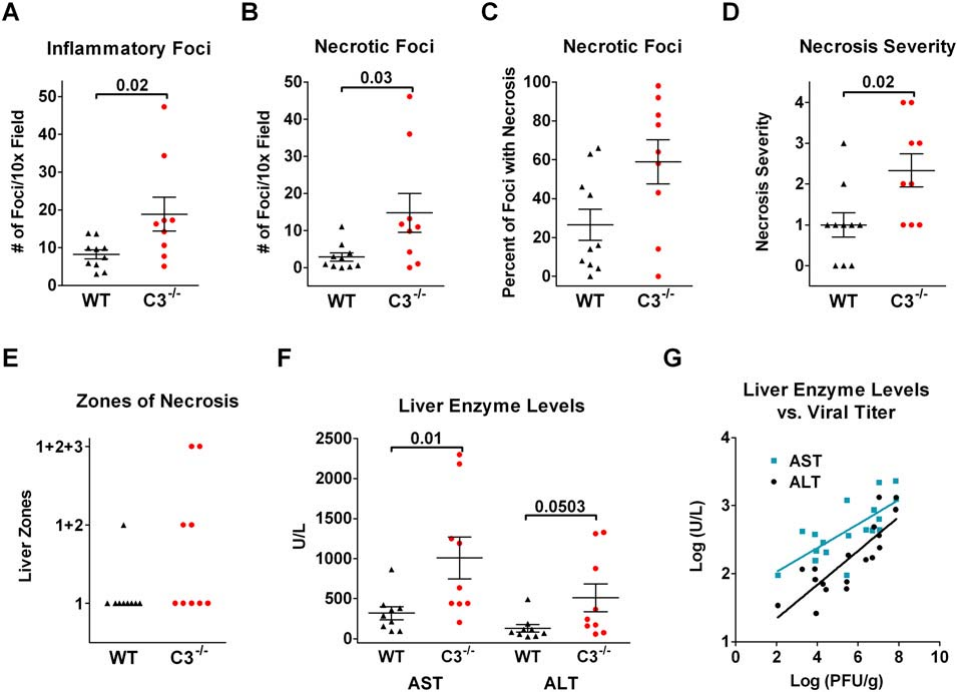
C3-deficient mice had a greater number of inflammatory foci with more extensive necrosis. (A–F) Wild-type (WT, black, ▴) and C3^−/−^ (red, •) mice were compared, and *P* values are above the bracket. Error bars are SEM. (A) The number of inflammatory foci/visual field was counted. Each point represents the mean from ∼7 fields for an individual mouse. The graph plots the mean number of foci for each animal. (B) The number of inflammatory foci containing necrosis was counted and displayed as described in (A). (C) The percentage of foci that contained areas of necrosis. (D) The severity of the necrosis was quantitated using a 0–4 scale: 0, none; 1, piecemeal necrosis; 2, confluent areas of necrosis; 3, confluent areas of necrosis that extend beyond a single zone; and 4, bridging necrosis. (E) The zones of the liver (1, 2, and 3) where necrosis occurred. Zones are described in Figure 3A. (F) The serum levels of the liver enzymes in uninfected mice were <100 for AST and <50 for ALT. (G) AST (blue) and ALT (black) levels positively correlate with liver viral titer (r^2^ = 0.54 and 0.69, respectively). The log of these parameters for both C3^−/−^ and wild-type mice was used for linear regression.

On day 4, the liver histopathology appeared normal in 4 of 5 wild-type and 3 of 4 C3^−/−^ mice (data not shown). By day 7, all animals had a diffuse lymphocytic infiltrate in addition to discrete inflammatory foci (Figure 3). These lesions varied in size and were smaller and less frequent in the wild-type (Figure 3A and 3B) compared to the C3^−/−^ mice (Figure 3C and 3D). They often occurred near the portal triad, and some contained areas of coagulative necrosis. An inflammatory infiltrate encircled the discrete necrotic foci (Figure 3B and 3C) and bordered the areas of bridging necrosis (Figure 3D). In contrast to the liver, no major differences were observed in the spleen at this time (data not shown). Using blinded samples, we counted the necrotic and non-necrotic foci and evaluated the location and severity of the necrosis in the liver (Figure 4).

**Figure 5:**
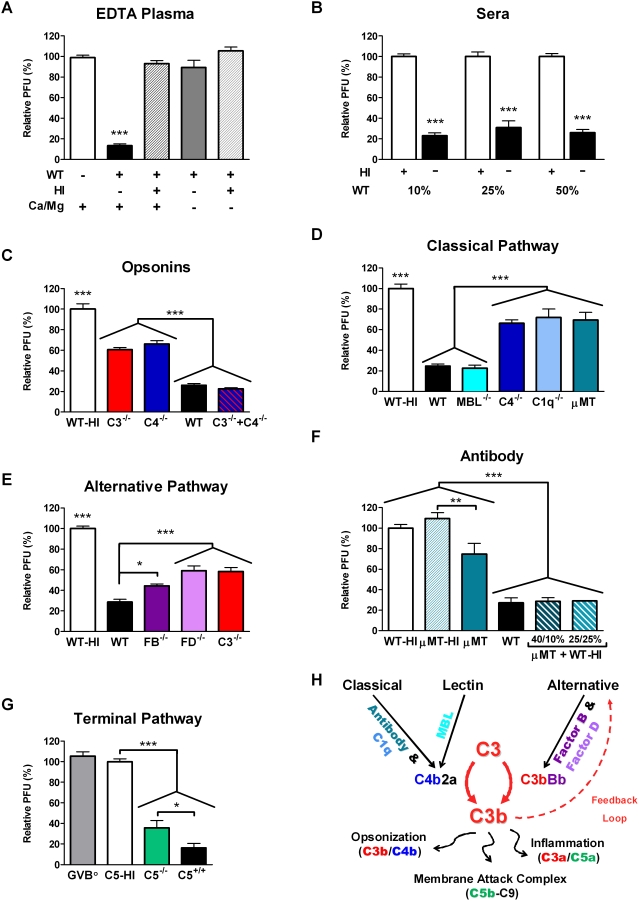
The murine complement system neutralized ECTV virions. (A–G) ECTV was incubated with a mouse complement source at 37°C for 1 hour and then added to BSC-1 monolayers. The absolute number of plaques was normalized to the appropriate controls (white bars) to give the relative number of plaques. Graphs display mean±SEM from multiple experiments. All data were analyzed using 1-way ANOVA followed by the Tukey multiple comparison test except (B), which used a 2-way ANOVA to analyze concentration and activity. (A) Mouse plasma neutralizes ECTV. ECTV was combined with 10% wild-type (WT) mouse EDTA-treated plasma or heat-inactivated (HI) plasma in gelatin veronal buffer (GVB) in the presence or absence of calcium and magnesium (Ca/Mg). The active plasma (in the presence of Ca/Mg) differed from all other groups (***, *P*<0.001). These data were combined from nine independent experiments using EDTA plasma collected on three separate days. The total number of replicates for each column from left to right follows: *n* = 25, 29, 17, 7, 3. (B) Mouse serum neutralizes ECTV. ECTV was incubated with increasing concentrations of HI (white) or active (black) WT mouse sera. Data represent three to four independent experiments performed in duplicate with four different collections of sera. Active sera neutralized ECTV (***, *P*<0.0001), at multiple concentrations. (C–G) The relative roles of the complement pathways were analyzed using sera from mice genetically deficient in complement components (C3, C4, C1q, MBL A and MBL C, FB, FD, or C5) or antibody (μMT). A final concentration of 50% sera was used in all cases. Data were combined from at least two experiments performed in duplicate with independent collections of sera. Unless noted specifically below, the significant differences are displayed on the graph as follows: *, *P*<0.05; **, *P*<0.01; ***, *P*<0.001. Any significant differences among grouped bars are noted on the graph. (C) C3^−/−^ or C4^−/−^ sera have reduced neutralizing capacity. Combining C3^−/−^ and C4^−/−^ sera restored neutralizing activity to WT levels. WT-HI differed from the other conditions (***). Data were from at least three experiments performed in duplicate. (D) Deficiencies in the classical pathway (C4, C1q) and antibody (μMT), but not the lectin pathway (MBL), reduce neutralization. WT-HI differed from C1q^−/−^ (*P*<0.01) and differed from the rest of the conditions (*P*<0.001). Data were from at least two experiments performed in duplicate. (E) Neutralizing activity requires the alternative pathway components: FB, FD, and C3. WT-HI differed from the other conditions (***). Data were from at least two experiments performed in duplicate. (F) Addition of either 10% or 25% WT-HI sera restores the neutralizing activity of antibody-deficient μMT sera to WT capacity. Heat inactivation of μMT sera reduces neutralization. Data were from two experiments done in duplicate. (G) MAC (C5b-9) formation enhances neutralization but is not required. Both the C5^+/+^ and C5^−/−^ sera had greater neutralization than HI-C5^+/+^. The buffer only control (GVB°) is equivalent to WT-HI. Data combined from three experiments performed in triplicate. (H) The classical, lectin, and alternative pathways each initiate the complement cascade by forming a C3 convertase that cleaves C3 to C3b. Antibody triggers the classical pathway through C1q. The carbohydrate motifs on pathogens activate the lectin pathway through MBL. The classical and lectin pathways form the C4 containing convertase, C4b2a. C3 deposited from these two pathways or from spontaneous activation of C3 initiates the alternative pathway. Upon binding, the activated C3 molecule FB is cleaved to Bb by FD, which forms the alternative pathway convertase, C3bBb. The alternative pathway amplifies C3b deposition by any pathway. C3 activation leads to important antiviral effector functions. Release of the anaphylatoxins C3a and C5a recruits inflammatory cells. C3b and C4b opsonize viral particles or infected cells, leading to neutralization or destruction by phagocytosis. C3b also leads to activation of C5 and formation of the MAC which disrupts virions or infected cells.

There were prominent differences between the C3^−/−^ and wild-type mice relative to the number inflammatory foci and in the degree of necrosis. The C3^−/−^ mice had twice as many total foci (8 vs. 18 per field, *P* = 0.02, Figure 4A) and 5-fold more foci containing regions of necrosis (3 vs. 15 per field, *P* = 0.03, Figure 4B). The majority of inflammatory foci contained necrotic areas in two-thirds of the C3^−/−^ mice compared to only one-fifth of the wild-type mice (Figure 4C). The C3^−/−^ mice had larger foci with more extensive necrosis (*P* = 0.02, Figure 4D). Most wild-type mice had small foci with either no necrosis or only piecemeal necrosis (0 and 1 on necrosis severity scale, Figure 3A and 3B, respectively). In contrast, the C3^−/−^ mice had confluent areas of necrosis that coalesced into bands of bridging necrosis (2 and 4 on the necrosis severity scale, Figure 3C and 3D, respectively). Given that necrosis most frequently occurred in zone 1 of the liver, it likely originated there and then extended into zones 2 and 3 (Figure 4E).

The increased hepatic necrosis in the C3^−/−^ mice resulted in higher levels of liver enzymes, aspartate aminotransferase (AST) and alanine aminotransferase (ALT), in the serum on day 7 (*P* = 0.008, 0.0503, respectively, Figure 4F). The AST and ALT levels positively correlated with the viral burden (Figure 4G).

Most C3^−/−^ mice died between day 7 and 10 (Figure 1C). Two C3^−/−^ mice that were sacrificed on day 10 had ∼5–7 inflammatory foci per field, while the 5 wild-type mice had only occasional foci (data not shown). At this time point, infectious ECTV persisted in the C3^−/−^ mice; whereas, wild-type mice had cleared the infection (Figure 2D).

### Mouse Complement Neutralized ECTV Intracellular Mature Virions

To explore the interaction between C3 and ECTV in vivo, we examined how mouse complement affects ECTV virions in vitro. Purified intracellular mature ECTV was incubated with either EDTA-treated plasma or sera from naïve C57BL/6 mice. Infectious virus was detected as plaques on a BS-C-1 monolayer. EDTA-treated plasma was reconstituted with a buffer containing calcium and magnesium to allow for complement activation.

Reconstituted wild-type plasma neutralized approximately 90% of the virus (Figure 5A, *P*<0.001). Heat inactivation or buffer lacking calcium and magnesium abolished neutralization. Wild-type sera concentrations of 10, 25, or 50% neutralized 70–80% of the ECTV (Figure 5B, *P*<0.0001). These observations implicate the complement system in neutralizing ECTV.

To further define if complement neutralized ECTV, sera from mice genetically deficient in a complement component or antibody were used in this assay (Figure 4C–4G). The neutralizing activity was reduced by ∼50% with deficiency of either C3 or C4 (Figure 5C). However, mixing C3^−/−^ and C4^−/−^ sera produced results equivalent to wild-type sera.

This requirement for C4 for full ECTV neutralization was further dissected. The C1q subunit of C1 interacts with antibody to trigger the classical pathway. MBL, a C1q analog, initiates the lectin pathway. MBL A^−/−^ x MBL C^−/−^, C1q^−/−^, and antibody-deficient (μMT) sera were compared (Figure 5D). μMT or C1q^−/−^ sera only partially neutralized ECTV, comparable to C4^−/−^ sera. Conversely, wild-type levels of neutralization occurred independent of MBL A and C. These data suggest that natural antibody activated the classical complement pathway to neutralize ECTV.

Further analysis revealed three key points relative to natural antibody. First, heat-inactivated wild-type sera behaves like buffer alone, which indicates that natural antibody alone lacks neutralizing activity; instead, complement activity was required to neutralize ECTV (Figure 5A and 5G). Second, heat-inactivated wild-type sera, as a source of natural antibody, restored the neutralizing activity of μMT sera (Figure 5F). Consistent with this finding, μMT or heat-inactivated wild-type serum did not effectively neutralize ECTV independently, but they did so in combination. Third, the modest but significantly greater neutralization in the normal compared to heat-inactivated μMT sera suggests that antibody-independent (alternate pathway) complement activation also occurred.

C3b deposited by any pathway interacts with factor B (FB) and factor D (FD) to generate the alternative pathway C3 convertase, which amplifies C3b production. Alternative pathway activation itself likely explains the neutralization observed in the μMT, C1q^−/−^, or C4^−/−^ sera. Interestingly, FB^−/−^ or FD^−/−^ sera neutralized less ECTV than wild-type sera (Figure 5E), which indicates that the alternative pathway enhanced complement-mediated neutralization initiated by the classical pathway.

C3b could neutralize ECTV by directly preventing attachment to or entry into the cell or by disrupting the virion's membrane through formation of the C5 convertase and the MAC. C5 initiates the terminal pathway that forms the MAC, and no lytic activity occurs in the absence of C5. C5^−/−^ sera from C57BL/10 mice were used to define the contribution of the MAC to neutralization (Figure 5G). C5^−/−^ sera neutralized a significant portion of virus (*P*<0.001), however, less than C5^+/+^ sera (*P*<0.05). These findings suggest that opsonization by C4b and C3b mediated most of the neutralization; although, the MAC also contributed.

To conclude, these findings demonstrate that naïve wild-type mouse sera neutralized ECTV. We propose that natural antibodies bound to ECTV and triggered the classical pathway. This led to C4b deposition, formation of the C3 convertase, and C3b deposition on the virus. The alternative pathway amplified the C3b placed on the virion by the classical pathway. Most ECTV neutralization occurred through opsonization by C4b and C3b, with a minor contribution from the MAC.

### Surviving ECTV Infection Required Multiple Complement Pathways

**Figure 6:**
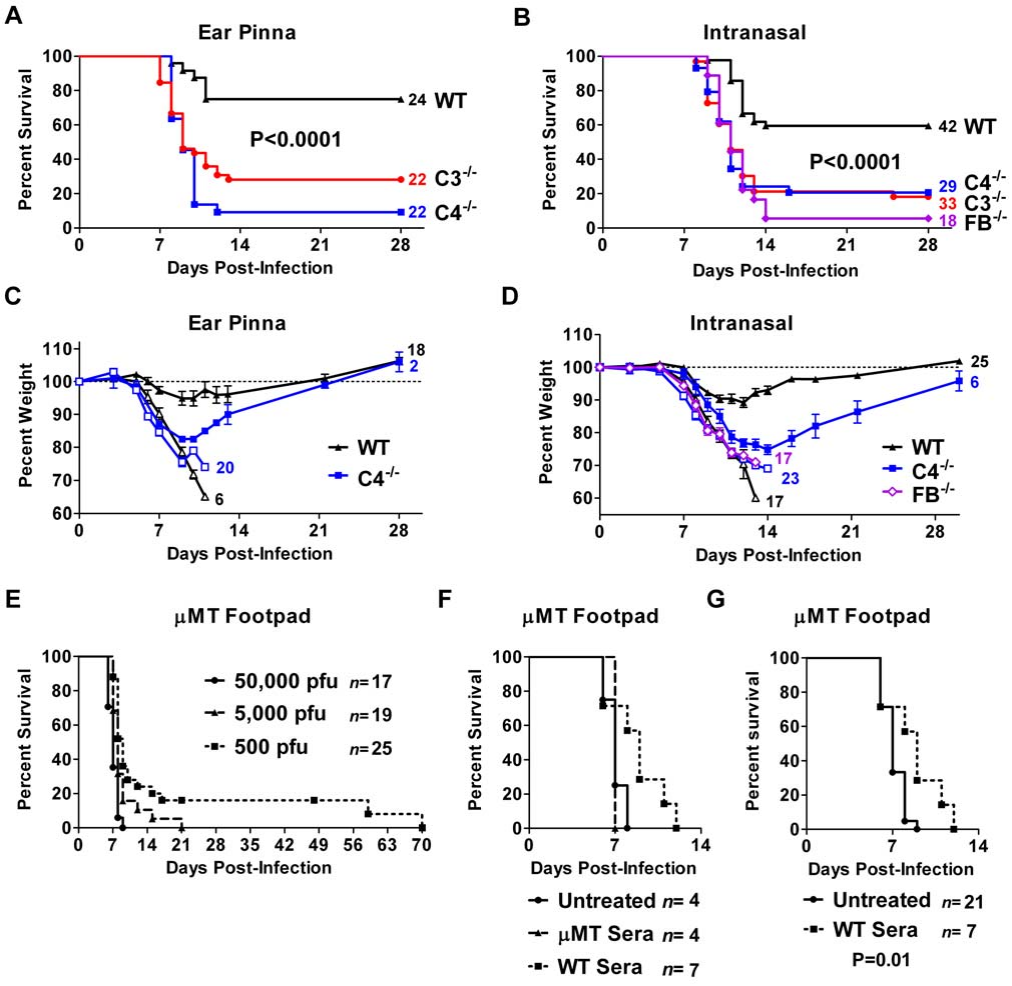
Deficiency of classical or alternative pathway components reduced survival. (A,C) C4^−/−^ mice have greater mortality than wild-type (WT, *P*<0.0001). C4^−/−^ mice were compared to C3^−/−^ and wild-type mice following inoculation with ∼700 pfu ECTV via the ear pinna. The weights of the surviving animals (filled symbols) are separated from those that succumbed (open symbols). The number of animals in each group is at the right of the line. The data combine four separate experiments. (B,D) Increased mortality in C4^−/−^, FB^−/−^, and C3^−/−^ mice compared to wild-type (*P*<0.0001). Mice received 100 pfu intranasally. Data are displayed as described above. Curves are generated from multiple experiments (*n* for WT = 8, C4 = 6, FB = 4, C3 = 7). The surviving FB^−/−^ mouse was omitted from (D) for clarity. (E) μMT mice are susceptible to ECTV via the footpad route. μMT mice were challenged with ECTV, and a dose-dependent increase in mortality was observed. The 50,000 pfu dose differs from 5,000 pfu (*P* = 0.006) and 500 pfu (*P*<0.0001), and the lower doses differ from each other (*P* = 0.045). Survival curves were constructed from four separate experiments, and the number of animals is to right of the legend. (F,G) Treating μMT mice with natural antibody delays mortality. μMT mice were challenged with high doses of ECTV (100,000 pfu) via the footpad route in two separate experiments. Some mice received wild-type sera as a source of natural antibody, while others received μMT sera (1 ml on day minus-1 followed by 0.5 ml on days 0, 2, 4, 6, 8, 10). The untreated curve in (G) includes the historical control data from the untreated mice at 50,000 pfu in (E). The number of animals in each group is next to the legend.

Both the classical and alternative pathways contributed to ECTV neutralization in vitro. To examine the importance of each pathway in vivo, we compared C4^−/−^ and FB^−/−^ mice to C3^−/−^ and wild-type mice. We challenged C4^−/−^ mice via the ear pinna route and monitored survival and weight loss. Over 90% of the C4^−/−^ mice succumbed to the infection (*P*<0.0001, Figure 6A). The C4^−/−^ and C3^−/−^ mice had comparable mortality and weight loss (Figures 6C and 1D).

Intranasal ECTV infection also produced similar results in the C3^−/−^, C4^−/−^, or FB^−/−^ mice. Each complement-deficient strain had a higher mortality rate compared the wild-type mice (*P*<0.0001), and there were no significant differences among the three strains (Figure 6B). The complement-deficient strains also lost weight at a similar rate (Figures 6D and 1F). Thus, control of ECTV in vivo required both the alternative and classical pathways, analogous to the in vitro results.

### Natural Antibody Delayed Mortality in μMT Mice

Complement poses a barrier to the systemic spread of pathogens, particularly through the bloodstream [17]. The major role of complement could be to neutralize ECTV recognized by natural antibody. Our prior experiments established that B cell-deficient μMT mice challenged with a high dose of ECTV by the footpad route all died early in infection (94% by day 8) (Figure 6E). Their early death suggests that B cells contribute to survival prior to the rise of specific antibody on day 7 [5].

Based on our in vitro data and the data of others [60],[61], we hypothesized that natural antibody contributes to early protection. Consequently, providing μMT mice with natural antibody should prolong their survival. Based on the work of *Ochsenbein et al.* [61], μMT mice infected with a high dose of ECTV were treated with naïve sera from either μMT or wild-type mice (Figure 6F). Treatment with wild-type sera increased the median day of death from 7 to 9; however, sera lacking natural antibodies (μMT) had no effect. On day 8 post-infection, over half of the mice receiving wild-type sera outlived both other groups and 16 of 17 mice from the prior experiments (Figure 6G). Thus, natural antibody delayed, but did not prevent, lethal ECTV infection in μMT mice.

## Discussion

We investigated the impact of complement deficiency using the ECTV mouse model. Deficiency of C3, C4, or FB resulted in acute lethal infection, establishing a requirement for multiple complement pathways in host defense against this pathogen. Specifically, C3 deficiency permitted ECTV to disseminate earlier, reach a higher titer in the target organs, and induce greater liver damage. Consistent with these in vivo results, naïve mouse sera neutralized ECTV infectivity in vitro, and sera lacking either classical or alternative pathway components had decreased activity. Several lines of evidence indicate that natural antibody initiated the classical complement cascade in the wild-type mouse. Substantial neutralization occurred in sera without lytic activity, which points to opsonization as the predominant mechanism of neutralization. Based on these results, we propose that natural antibody binds viral antigen to activate the classical pathway, followed by engagement of the alternative pathway's feedback loop to opsonize the virus.

The ECTV model system provides several advantages for analyzing the role of complement in poxviral pathogenesis. First, the mouse-specific pathogen ECTV has coevolved with and causes severe disease in the natural host, analogous to variola virus in humans. Second, the role of complement and the pathways involved can now be more rigorously dissected in vivo and in vitro with the availability of complement-deficient mice. Additionally, the in vitro experiments employed sera from the same strains used to characterize the effect of complement deficiency in vivo, and the neutralizing capacity in vitro paralleled the in vivo mortality. Third, viral pathogenesis, morbidity, and mortality can be assessed by multiple routes of infection and across a range of viral inoculum to demonstrate a broad requirement for complement.

Complement-deficient mice succumbed to acute ECTV infection with the majority of deaths occurring between days 6–10. Based on time to death following footpad inoculation, C3 deficiency resembled immunodeficiencies of other important components of the antiviral response, specifically CD8^+^ T cells [6],[7], NK cells [9],[10], and IFN-γ [12]. In contrast, mice deficient in CD4^+^ T cells [6], CD40, or CD40 ligand (CD154) [7] survive the acute phase but do not clear the virus. The CD40^−/−^ and CD154^−/−^ mice ultimately die ∼4 to 8 weeks post-infection. This differs from surviving C3^−/−^ mice, which recovered and did not show signs of ongoing illness for up to 4 months of observation. The early death of the complement-deficient mice highlights the complement system's essential contribution to survival during the first few days of infection.

To characterize how complement protects the host from lethal infection, we analyzed the impact of C3 deficiency on the kinetics of viral spread. ECTV replication at the inoculation site and in the draining lymph node produces a viremia that seeds the primary target organs, the liver and spleen [4]. Several observations from this study increase our understanding of complement's role in controlling poxviral infection.

First, as early as day 2, C3 deficiency allowed for greater spread of ECTV from the inoculation site to the spleen. This indicates that complement is a key player in the initial hours of infection, likely to control ECTV at the inoculation site. Second, we detected higher levels of viral DNA in the blood on days 4 and 7. Consistent with our in vitro data, these results establish that C3^−/−^ mice poorly control viral dissemination through the bloodstream. This higher viremia could result from increased replication in tissues and/or decreased clearance of virus from the bloodstream. Third, the liver viral titers on day 7 were ∼50-fold higher in the C3^−/−^ mice. The greater viremia likely produced more extensive infection, but a delayed adaptive immune response may also have contributed to this observation. The viral titer correlated with serum levels of ALT, which suggests that ECTV caused hepatic necrosis either directly through lytic infection or indirectly through the antiviral immune response. An inflammatory infiltrate surrounded the necrosis in the C3^−/−^ mice, which contrasts with susceptible Balb/c mice where necrosis occurs in the absence of a lymphocytic infiltration [62]. In summary, we propose that mice lacking C3 have reduced ability to control ECTV locally and in the bloodstream, leading to higher levels of infection and greater tissue damage in the liver.

Complement could delay viral dissemination by opsonizing and thereby neutralizing virions at the inoculation site or in the circulation and by promoting the inflammatory response including the recruitment of phagocytic cells. To assess if complement could directly neutralize ECTV, we examined the interaction between purified ECTV and mouse complement in vitro. Naïve plasma or sera neutralized ECTV in a complement-dependent manner, even at a concentration as low as 10%. Sera from mice deficient in specific complement components demonstrated that maximal neutralization required both the classical and alternative pathways. μMT sera, lacking antibody, resembled the sera deficient in the classical pathway components, C1q or C4, and addition of a natural antibody source restored neutralization activity. Opsonization led to neutralization of the majority of virus; however, the modest but significant difference between the C5^−/−^ and C5^+/+^ sera indicates that the MAC contributed to viral damage. Interestingly, no complement component deficiency tested fully abolished neutralization. The residual activity suggests that the classical and alternative pathways functioned independently, likely because both C4b and C3b opsonized and, consequently, neutralized ECTV. However, the system was most effective when the two pathways and the MAC worked cooperatively.

The reconstitution of the neutralization activity in the μMT sera with heat-inactivated wild-type sera suggests that natural antibody is important in the neutralization process. Consistent with this observation and prior studies with other viruses [60],[61], natural antibody passively transferred into μMT mice lengthened survival during the acute infection. In our experiments, most μMT mice died early, with 100% mortality by day 9 at the highest inoculum. The mice that survived the acute infection eventually died at ∼2 months post-infection. Our findings differ from prior studies, which described mortality at either 2–4 weeks [8] or 2 months [7] post-infection. More of our mice survived the acute infection at the lower doses. This suggests that the observed discrepancy could be secondary to differences in the viral stock or dose, as both differed among the three groups. The death of the μMT mice, despite natural antibody treatment, indicates that B cells help control the infection by additional mechanisms.

Our in vitro experiments provide a model for understanding the fate of the viral inoculum in our in vivo experiments, since they both used the same stock of purified intracellular mature virus (IMV). To understand the spread of infection, a second infectious form must be considered. During viral replication in the host cell, extracellular enveloped virus (EEV) is produced by enveloping the IMV with an additional unique membrane derived from the Golgi complex and late endosomal compartment [63]. In studies of vaccinia virus, the host's complement regulators, present in the outermost membrane, protect the EEV from human and rabbit complement; in contrast, the IMV is sensitive to complement [64]. Our study builds on this observation by determining the contribution of each complement activation pathway to the neutralization of IMV infectivity, and it implicates natural antibody as the primary initiating factor [61]. We also show that natural antibody by itself is ineffective but requires augmentation by the complement system to neutralize ECTV. Additionally, the neutralization observed with vaccinia virus and ECTV points to the IMV form being inherently susceptible to complement-mediated neutralization.

The relative importance of IMV vs. EEV during infection in vivo is not well established. However, the IMV's sensitivity to complement neutralization suggests that ECTV likely travels through areas featuring efficient complement activation, such as the blood stream, in the EEV form or within infected cells. At extravascular sites, where complement levels are lower than in circulation, infected cells may produce sufficient soluble poxviral complement regulatory protein to protect the IMV.

Most poxvirus disease models initiate infection with the complement-sensitive IMV. If complement activity in the mouse behaves as it does in vitro, then inoculated ectromelia IMV should be recognized by natural antibody and coated with C4b and C3b, resulting in neutralization of viral infectivity at the site of injection and inhibition of spread. This line of reasoning could explain why the mortality increases in the wild-type mice as the invasiveness of the route decreases [52]. Percutaneous inoculation would likely result in neutralization, while application to the mucosal membranes might enable ECTV to enter host cells before being neutralized by complement. Once internalized, ECTV produces its regulatory protein and EEV to evade complement and propagate the infection. Additionally, based on the in vitro data, complement deficiency would greatly limit this initial neutralization, which likely contributes to the early spread and greater mortality observed in the complement-deficient mice.

A sub-neutralizing concentration of complement opsonins could target the virion for immune adherence and phagocytosis in vivo, particularly in blood with its high complement levels. Furthermore, the liver sinusoids are lined with Kupffer cells bearing CRIg (Complement Receptor of the Ig superfamily), which mediates phagocytosis of C3-opsonized pathogens [65]. Indeed, the liver clears over 95% of intravenously administered ECTV from the circulation within 5 min of injection [66]. In the following hour, most of the viral antigen in the liver becomes undetectable by immunofluorescence, and viral infectivity decreases by over 90%. This rapid removal suggests that the virus has been recognized as foreign and tagged for immune adherence and destruction. Opsonization by complement followed by uptake via the recently described CRIg provides a mechanistic explanation for these important observations made nearly five decades ago [66].

These observations influence the interpretation of poxviral infections initiated with an IMV-rich inoculum by the intravenous route. The liver's Kupffer cells may sequester most of the inoculated virus within minutes and destroy much of it within an hour, thereby inhibiting systemic dissemination. Not only is the dose effectively reduced by ∼10-fold, but the neutralized IMV also provides the immune system with an immediate source of antigen. These issues have particular relevance for the monkeypox and variola virus non-human primate models that commonly use the intravenous route to test vaccines for human use [67]–[72].

The early mortality of the C3^−/−^, C4^−/−^, and FB^−/−^ mice demonstrates an essential role for the classical and alternative pathways in the initial stages of poxvirus infection. Despite equivalent mortality levels, further analysis may reveal different functions for each complement pathway in vivo, as such differences exist in the immune response to other viruses [23]. The similarity between the in vivo mortality and in vitro serum neutralization experiments suggests that complement neutralizes ECTV and thereby limits its spread. Undoubtedly, complement deposition triggers other effector functions, such as recruiting inflammatory cells, promoting phagocytosis, and priming the adaptive immune response. The precise contribution of each of these to protection in vivo remains unexplored.

However, these experiments establish that complement is essential to the immune response to poxviruses. It accounts for why the virus encodes a potent complement regulatory protein. A virus lacking this regulator would be at risk for greater host complement activity and attenuation. This is consistent with the theory that loss of this regulator contributes to the reduced virulence of some strains of monkeypox virus [46]. To conclude, the complement system is critical for slowing down viral spread and decreasing tissue titers and damage.

## Materials and Methods

### Virus Production

Plaque-purified Moscow strain ECTV was propagated in murine L929 cells. Intracellular mature viral stocks were purified through a sucrose cushion as described [73] and titrated on BS-C-1 cells, an African green monkey kidney cell line [74]. Both cell lines were cultured in Dulbecco's modified Eagle's media (DMEM, BioWhittaker, Walkersville, MD) supplemented with 10% heat-inactivated fetal calf serum (FCS, HyClone, Logan, UT), 2 mM L-glutamine, and antibiotics.

### Mice 

The following strains on a C57BL/6 background were acquired: C3^−/−^ [56],[75] and FB^−/−^ [76],[77] from H. Molina, Washington University Medical School; C4^−/−^ [78] from M. Carroll, Harvard Medical School; B cell-deficient μMT [79] from H. W. Virgin, Washington University Medical School; C1q^−/−^ [80] from M. Botto, Imperial College School of Medicine; FD^−/−^ [81] from Y. Xu, University of Alabama, Birmingham; and MBL A^−/−^ x MBL C^−/−^ (B6.129S4-*Mbl1^tm1Kata^ Mbl2^tm1Kata^*/J) and wild-type from Jackson Laboratories. The C5^+/+^ and C5^−/−^ C57BL/10 mice (B10.D2-*Hc^1^ H2^d^ H2-T18^c^*/nSnJ, B10.D2-*Hc^0^ H2^d^ H2*-*T18^c^*/oSnJ) were also obtained from Jackson Laboratories. Age-matched mice of both sexes were used in the footpad and ear pinna studies (6–11 weeks-old) and the μMT survival experiments (10–11 weeks-old). Male mice were used in the intranasal (8–12 weeks) and sera transfer (10–12 weeks) studies. Some wild-type and μMT mice used in the footpad studies were purchased from Jackson Laboratories. The rest of the mice were bred at Washington University in a specific pathogen-free facility. The animals were transported to the biohazard suite at Saint Louis University at least a week prior to infection. All experiments were performed following the animal care guidelines of the two institutions.

### In Vivo Studies

Mice were inoculated with 10 µl ECTV diluted in PBS to the indicated dose using a 29 gauge insulin syringe into the ear pinna and hind footpad or a 20 µl pipettor for the intranasal route. Mice were anesthetized for inoculation using CO_2_/O_2_ for the footpad route and ketamine/xylazine for the ear pinna and intranasal routes. Individual mice were marked by ear punching or shaving. After infection and before sacrifice in the mortality studies, mice were manipulated only to obtain weights. Serum was collected from surviving animals at the end of the experiment. The survival curves include only animals that generated an antiviral antibody response, which was detected by ELISA in >95% of the mice [82]. In the passive transfer experiment, mice received intraperitoneally 1 ml of wild-type or μMT C57BL/6 sera on day −1 and 0.5 ml every two days starting on day 0.

### Tissue Titer

Blood was collected via cardiac puncture. Spleen and liver tissues were harvested aseptically, frozen immediately on dry ice, and stored at −70°C. Tissues were homogenized in PBS-1% FCS to ∼10% (weight/vol) using 1 ml glass homogenizers. They were frozen and thawed three times, sonicated, and titrated on BS-C-1 monolayers [74].

### Viremia Analysis

DNA was isolated from whole blood collected in EDTA microtainer tubes (BD, Franklin Lakes, NJ) using the High Pure PCR Template Preparation Kit (Roche). The kit's whole blood protocol was used with the following modifications. The 40 µl Proteinase K, 200 µl Binding Buffer, 150 µl PBS, and 50 µl of whole blood in EDTA were added sequentially and then vortexed. The incubation at 70°C was extended to 12 min. The sample was applied to the column by centrifugation at 8,000 *g* for 2 min and eluted in 50 µl.

Quantitative PCR was performed on viral DNA using Power SYBR Green PCR Master Mix on a 7500 Real Time PCR System (Applied Biosystems, Foster City, CA) [83]. The primers (10 pmol) SP028 (GTAGAACGACGCCAGAAT AAGAATA, 5′ at 120627 bp) and SP029 (AGAAGATATCAGACGATCCACAATC, 5′ at 120462 bp) were used to amplify 165 bp of gene EV107. The amplification product cloned into a plasmid vector (pGEM-T, Promega) was used as a standard to estimate copies of DNA/µl in blood. Three to four wells were used for each sample.

### Histology

Tissue samples were fixed in 10% buffered formalin, embedded in paraffin, sectioned, and stained with hematoxylin and eosin by the Digestive Diseases Research Core Center, Washington University. The number of inflammatory foci and the magnitude of tissue necrosis were evaluated in blinded samples. Inflammatory foci in a 10× visual field were counted for ∼7 fields/mouse liver.

### Liver Enzymes

AST and ALT levels were measured in samples of frozen sera by the Department of Comparative Medicine at Saint Louis University using a standard clinical analyzer.

### Complement Neutralization Assay

Mouse EDTA plasma and sera were collected on ice from male C57BL/6 mice in microtainer tubes (BD), separated by centrifugation, and then pooled, aliquoted, and frozen at −70°C. Plasma and sera were diluted on ice into GVB± Ca^++^/Mg^++^ (#B102, B103, Complement Technology, Tyler, TX) or GVB without Ca^++^/Mg^++^, respectively, to 2× the desired final concentration (vol/vol). Purified ECTV was sonicated and diluted in PBS (without Ca^++^/Mg^++^) to ∼5×10^4^ pfu/ml. A 1∶10 dilution in the buffer used to dilute the complement source, GVB±Ca^++^/Mg^++^, produced a final concentration of ∼5 pfu/µl. An equal volume of virus (30 µl≈150 pfu) was added rapidly to the diluted complement at RT. Samples were vortexed, centrifuged for 5 sec, and incubated at 37°C for 60–90 min. Samples were diluted by addition of 700 µl of DMEM-2% FCS, vortexed, and applied to BS-C-1 monolayers in 6-well plates. After 1 hr, 3 ml/well of 37°C overlay media (1% carboxymethylcellulose in culture media) was added. After 3–5 days, the cells were fixed with 1 ml/well of an 11% formaldehyde/ 0.13% crystal violet/ 5% ethanol solution for over 1 hr, rinsed, and dried. The number of plaques was scored visually using a light box. The EDTA plasma or sera data were normalized to the buffer only control or heat-inactivated sera, respectively.

### Statistical Analysis

All statistical analysis was performed using GraphPad Prism software version 5.01 (GraphPad Software, San Diego, CA). The survival curves were analyzed by the log-rank test. The Mann-Whitney test was used to determine the statistical significance of the viral titers, viremia, liver histology, and liver enzymes. Either 1-way ANOVA followed by Tukey multiple comparisons test or 2-way ANOVA was used for the analysis of the complement neutralization assays.

## Supporting Information

Figure S1 C3 deficiency promoted earlier dissemination to and increased viral titers in the target organs. C3-deficient and wild-type mice were infected with ∼700 pfu of ECTV via the ear pinna and sacrificed on day 2, 4, 7, or 10 post-infection. The viral titer of the spleen (A) and liver (B) was determined by direct plaque assay and is expressed as the log_10_ plaque forming units (PFU)/g tissue. The level of viral DNA in whole blood was measured using quantitative PCR (C). Mean viral titers±SEM or genome copies±SEM are plotted against time. The dotted line represents the limit of detection for the assay.
(0.49 MB TIF)
