# Supplementary figures and images for: Surviving Mousepox Infection Requires the Complement System
Source: PLoS Pathog. 2008 Dec 26;4(12):e1000249. doi: 10.1371/journal.ppat.1000249 (PMC2597719; doi:10.1371/journal.ppat.1000249)

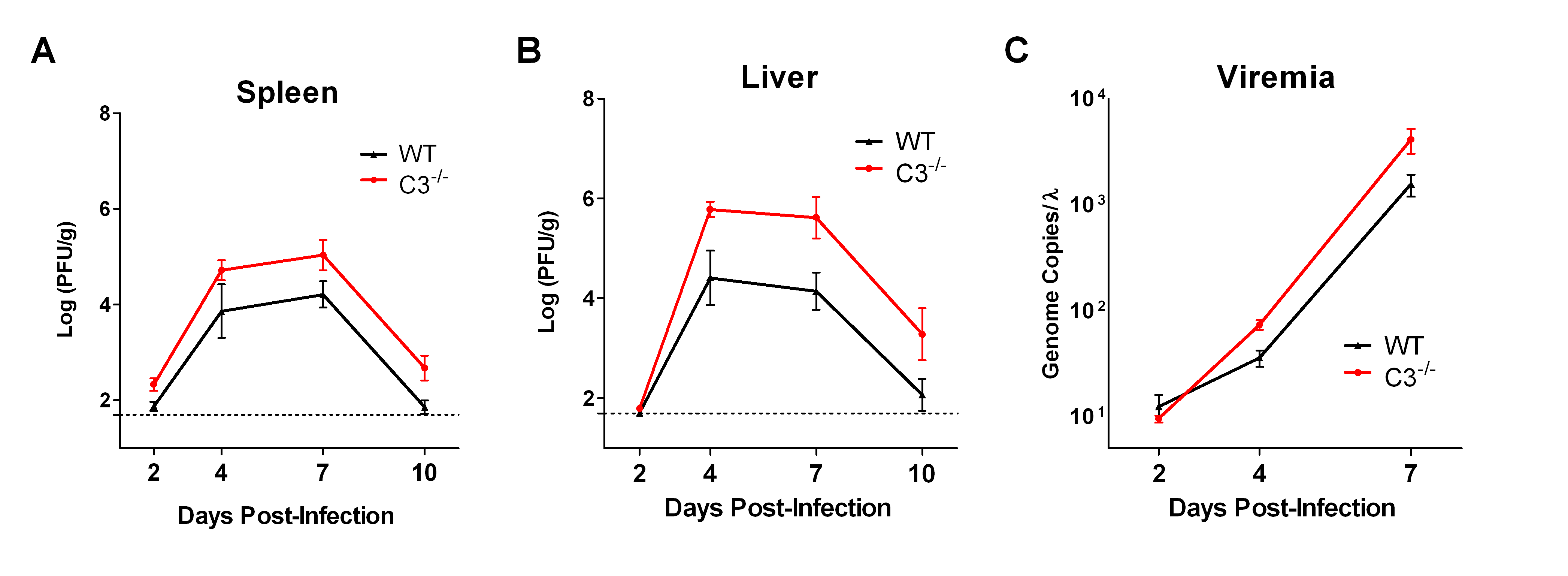

Supplement: Figure S1 — C3 deficiency promoted earlier dissemination to and increased viral titers in the target organs. C3-deficient and wild-type mice were infected with ∼700 pfu of ECTV via the ear pinna and sacrificed on day 2, 4, 7, or 10 post-infection. The viral titer of the spleen (A) and liver (B) was determined by direct plaque assay and is expressed as the log10 plaque forming units (PFU)/g tissue. The level of viral DNA in whole blood was measured using quantitative PCR (C). Mean viral titers±SEM or genome copies±SEM are plotted against time. The dotted line represents the limit of detection for the assay. (0.49 MB TIF) [file ppat.1000249.s001.tif]
